# Influence of COVID-19 on lifestyle behaviors in the Middle East and North Africa Region: a survey of 5896 individuals

**DOI:** 10.1186/s12967-021-02767-9

**Published:** 2021-03-30

**Authors:** Mohamed Abouzid, Dina M. El-Sherif, Nael Kamel Eltewacy, Nesrine Ben Hadj Dahman, Salah A. Okasha, Sherief Ghozy, Sheikh Mohammed Shariful Islam, EARG Collaborators, Akram Ramadan Farag Elburki, Almajdoub Ali Mohammed Ali, Moufiq Abdulrasul Hasan, Emadaldin Awad Amgrab ALI, Mahmoud N. Gumma Mohamed, Mareb H. Ahmed, Ayat K. Taher, Luma Saad Abdalbaqi, Nuha Hadi Jasim, Ibrahim Adel Aziz, Amna Babiker Dafaallah, Sirine Ben Dalla, Omar Mastouri, Chams Zarrad, Asmaa E. Abdelslam, Ahmed Taha A. Sayed, Kyrillos Wassim, Mahmoud Shaban, Tarek Mohamed Fayez, Mohammed Nasser, Zahraa Radhi, Zahraa Alkuwaiti, Amina Alsaffar, Asmaa M. Elaidy, Majeda Hammoud, Sabri M. Hammoud, Ali Saheb Alturki, Tasneem Jarkhi, Rama Hamza, Nourelhuda Mahmoud Issa, Reham Ahmad Kaakeh, Omar Alfatohi Aljondi, Ahmed Ali Ahmed, Mona H. Ibrahim, Islam Samy, Fatima Abdulrahman, Walaa Sabry Fouad, Normala Shahin, Saif Salman, Sumaia Ghunaim, Sarah Elayyan, Farah Ahmed Alkhaled, Deena Elsori, Samy Nael Altayeb, Elias Edward Lahham, Afnan W. M. Jobran, Asil Nael Salman, Aroub Imad Abdelhaq, Ola Hassan Akkawi, Ali Hassan Salah Al-Hadi, Sadeq Nagi Rashed Gozelan, Hatem Sultan Ahmed Qasim, Abdullah Ahmed Areqi, Azal Abduulah Abdurab Thabet, Boutheyna Drid, Sara Menzer, Melissa Hamdani, Zighed Alya, Najlae Adadi, Zakaria El Hamri, Hinde El Mouhi

**Affiliations:** 1 Department of Physical Pharmacy and Pharmacokinetics, Faculty of Pharmacy, Poznan University of Medical Sciences, Poznan, Poland; 2 National Institute of Oceanography and Fisheries, NIOF, Cairo, Egypt; 3 Faculty of Pharmacy, Beni-Suef University, Minia, Egypt; 4 Faculty of Medicine of Tunis, University of Tunis El Manar, HOD Medical Research; Doctors of the World Tunisia, Tunis, Tunisia; 5 Department of Agronomy, Plant Breeding and Bio-Statistic, Faculty of Agriculture, Suez Canal University Ismailia, Mansoura, Egypt; 6 Faculty of Medicine, Mansoura University, Mansoura, Egypt; 7 Institute for Physical Activity and Nutrition (IPAN), School of Exercise and Nutrition Sciences, Deakin University, Melbourne, Australia

**Keywords:** COVID-19, Coronavirus, Lifestyle, MENA

## Abstract

**Background:**

Coronavirus disease (COVID-19) pandemic has affected health and lifestyle behaviors of people globally. This project aims to identify the impact of COVID-19 on lifestyle behavior of individuals in the Middle East and North Africa (MENA) region during confinement.

**Methods:**

We conducted an online survey in 17 countries (Egypt, Jordan, United Arab Emirates, Kuwait, Bahrain, Saudi Arabia, Oman, Qatar, Yemen, Syria, Palestine, Algeria, Morocco, Libya, Tunisia, Iraq, and Sudan) from the MENA region on August and September 2020. The questionnaire included self-reported information on lifestyle behaviors, including physical activity, eating habits, smoking, watching television, social media use and sleep before and during the pandemic. Logistic regression was performed to analyze the impact of COVID-19 on lifestyle behaviors.

**Results:**

A total of 5896 participants were included in the final analysis and 62.8% were females. The BMI of the participants was 25.4 ± 5.8 kg/m^2^. Around 38.4% of the participants stopped practicing any physical activities during the confinement (P < 0.001), and 57.1% reported spending more than 2 h on social media (P < 0.001). There were no significant changes in smoking habits. Also, 30.9% reported an improvement in their eating habits compared with 24.8% reported worsening of their eating habits. Fast-food consumption decreased significantly in 48.8% of the study population. This direct/indirect exposure to COVID-19 was associated with an increased consumption of carbohydrates (OR = 1.09; 95% CI = 1.02–1.17; P = 0.01), egg (OR = 1.08; 95% CI = 1.02–1.16; P = 0.01), sugar (OR = 1.09; 95% CI = 1.02–1.16; P = 0.02), meat, and poultry (OR = 1.13; 95% CI = 1.06–1.20; P < 0.01). There was also associated increase in hours spent on watching television (OR = 1.07; 95% CI = 1.02–1.12; P < 0.01) and social media (OR = 1.09; 95% CI = 1.01–1.18; P = 0.03). However, our results showed a reduction in sleeping hours among those exposed to COVID-19 infection (OR = 0.85; 95% CI = 0.77–0.94; P < 0.01).

**Conclusions:**

The COVID-19 pandemic was associated with an increase in food consumption and sedentary life. Being exposed to COVID-19 by direct infection or through an infected household is a significant predictor of amplifying these changes. Public health interventions are needed to address healthy lifestyle behaviors during and after the COVID-19 pandemic.

**Supplementary Information:**

The online version contains supplementary material available at 10.1186/s12967-021-02767-9.

## Introduction

On December 31, 2019, cases of unexplained pneumonia were reported in Wuhan city, China [1]. After performing extensive investigations, isolation of a virus related to the genus coronaviruses was done and later named the novel coronavirus (COVID-19) by the world health organization on 12 January [1]. Soon afterward, COVID-19 disease turned into a global pandemic, affecting more than 71 million people worldwide by December 11, 2020 [2, 3]. Due to the massive number of infected people, several governments in the Middle East and North Africa (MENA) region announced more stringent containment measures for containing COVID-19 spread, including a general lockdown by the end of March 2020 for at least two weeks [2]. The MENA region has its social specificities, as group activities and social interaction are highly valued compared to other areas. Therefore, lifestyle and habits have changed due to social distancing and self-isolation, which are both considered strongly impacting the individuals’ lives during the pandemic.

Among the multiple consequences of the current pandemic, there have been two significant impacts; stockpiling food as a result of grocery restriction and spending more time indoor; including working from home, tele-education, and restricted outdoor physical activities [4–6]. Furthermore, quarantine work routine disruption could contribute to boredom which is linked to higher greater energy intake [7]. Besides, the frequent stressful exposure to visual and auditory news concerning COVID-19 can be linked to overeating, in particular high-sugar foods, known as “food craving” [8]. These habits may temporarily ease stress and give a false feeling of happiness since simple carbohydrates can influence the production of serotonin; hence, impact the mood positively [9]. Nerveless, there is a proportional relationship between carbohydrate food craving and food glycemic index that is associated with a higher risk of developing cardiovascular diseases, obesity, and chronic inflammation. Additionally, these diseases have been proven to raise the risk for more COVID-19 severe complications [10, 11].

In the same context, sedentary habits attributable to lockdown measures as alternations in sleeping, and smoking habits are substantially changing the lifestyle, especially among health workers [12]. A study conducted on 955 men showed that sleep could be a risk factor for obesity, especially in young men [13]. Regarding smoking, Berlin et al. have reported that mental distress and social isolation may lead to an increase in the need for smoking. During the lockdown, smoking will have a higher chance to impact second-hand smokers [14]. To date, there is no comprehensive global survey aiming to investigate the implications of COVID-19 lockdown on lifestyle behaviors in the MENA region. To address this gap, we conducted the current study to identify COVID-19 impacts on the physical activity levels and eating habits among individuals residing in the MENA countries.

## Methods

### Study design

A cross-sectional study was carried out using a self-administered structured online survey tool through the “Google Forms” platform.

### Study population

The inclusion criteria were all individuals who agreed to participate in the study, aged  ≥ 18 years, and living in one of the MENA region countries. There were no restrictions on gender, nationality, occupation, or socioeconomic level of the participants. The exclusion criteria were all residents less than 18 years and those who refused to take part in the study.

### Sampling

All participants fulfilling the inclusion criteria were invited to participate. The sample size was calculated according to the specific country setting for this multinational study. Snowball sampling will be used to select the study participants.

### Data collection

An online link of the web-based questionnaire was developed using “Google Forms” obtaining lifestyle data during August and September 2020. On the first screen, a Plain Language Information Statement (PLIS) and Consent Form were placed. Details of the local country-specific investigators will be included in the PLIS, who will be able to respond to any relevant queries during data collection. Only the participants providing consent to participate in the study can move to the next section containing the screening questionnaire to confirm the age is consistent with the pre-defined criteria. Upon confirmation, the participants were moved to the next pages containing the self-administered questionnaire.

The survey covered 17 countries from the MENA region: Egypt, Jordan, United Arab Emirates, Kuwait, Bahrain, Saudi Arabia, Oman, Qatar, Yemen, Syria, Palestine, Algeria, Morocco, Libya, Tunisia, Iraq, and Sudan. The collaborators were responsible for the distribution of the survey among the countries mentioned above via online platforms and social media (Twitter, Facebook, and Instagram) that are accessible through smartphones, laptops, and computers. Each country has been targeted by its collaborators to guarantee the efficacy and accuracy of survey filling. Using social media was effective because according to the latest report on internet usage in the MENA region, 70.2% of the population were connected to the internet and 51% were using social media [15].

### Questionnaire formulation and validation

Prior to developing the questionnaire, an extensive review of the relevant literature was conducted, followed by a discussion with experts on lifestyle and physical activity. Following the development of the first version of the questionnaire, it was validated by a panel of experts for face, content, criterion and construct components. Different reliability measures were also tested; including test–retest reliability/repeatability (Pearson correlation), internal consistency (Cronbach's alpha), and inter-rater reliability (Additional file 1).

For the validation purposes, a pilot study of 30 participants was performed. As for languages used, we formulated the survey in the English language then forward translation into Arabic, followed by a backward translation was done by two independent translators. The translations were finally reviewed by a team of investigators and translators to resolve any discrepancies.

### Study tool

The final format of the survey tool consisted of 36 questions, which were divided into three different sections: (1) personal data (11 questions: age, gender, country, height, weight, social status, resident region, occupation, education level, COVID-19 history, disease history); (2) daily eating habits (15 questions: daily eating routine, changes in weight, consumption of certain foods such as fruits and vegetable, carbohydrates, chicken and meat, fish and seafood, water, dairy, eggs, sugar, snacks, fast-food, drinks (caffeinated, carbonated and juice), and dietary supplements); (3) lifestyle (10 questions: smoking, hours spent on certain activities such as sleeping, sport exercising, television, social media, internet and family quality time). The entire questionnaire and scale are available in Additional file 1.

### Statistical analysis

Data were analyzed using IBM SPSS for Windows version 26 statistical software. Categorical data were reported as frequency/percentage and continuous data as mean/standard deviation. Continuous data were explored for normality by checking the distribution of data and using tests of normality (Kolmogorov–Smirnov and Shapiro–Wilk tests). Between-group comparisons were analyzed by one-way ANOVA [16]. Post hoc comparisons were performed using Duncan's test at significant levels of (P-Value ≤ 0.05). When application conditions were not met, Levene's test was used to test the homogeneity of variance. Paired sample t-test was used to compare the two scores (before and during) for each item. Moreover, binary regression was performed to study the impact of COVID-19 infection on lifestyle patterns. Logistic regression results were presented as odds ratios (ORs) and 95% confidence interval (95% CI).

## Results

### Participants

On the 4th of September 2020, the web-survey was concluded, and the collected data were analyzed. A total of 6019 participants completed the questionnaire, and, after validation, 5896 respondents were included. The participants were aged between 8–23 years (45%; 24–30, 20.5%; 31–40, 18.6%; 41–60, 13.6%; > 40, 2.3%) and 62.8% were females. The mean BMI was 25.4 ± 5.8 kg/m^2^. Territorial coverage spreads into various regions, 81.4% were located in the city, 19.9% in the countryside, 5.5% in coastal and 2.2% in the desert. Despite the small percentage of the participants in desert areas, their BMI was significantly higher compared with the city and coastal (P = 0.012, P = 0.021; respectively). In terms of employment status, 2680 (45.5%) were students, 1391 (22.4%) had a full-time job, 676 (11.5%) were working in the medical field, 566 (9.6%) unemployed, and 309 (5.2%) shift employee. Most of the participants (62.3%) were single, 2094 (35.5%) were married, and 130 (2.2%) were divorced. Almost two-third of participants (68.5%) had higher education. 14.1% of the participants reported that they or any of their household members had been infected with COVID-19. Data from each country is shown in Additional file 2: Tables S1, S2, S3, S4.

### Lifestyle changes during COVID-19 emergency

Various habits have been significantly impacted during COVID-19, such as sleeping hours, physical activities, TV watching hours, and family quality time (Table 1). 49.6% reported they were sleeping less than 7 h and the sleeping hours increased to 7–10 h. for 53.2% (P < 0.001). Physical activity frequency has also significantly decreased (P < 0.001) during the COVID-19, 48% reported stopping physical activities during COVID-19 compared with 44.6% before the lockdown. Besides, the type of physical activity has also changed significantly, such as walking (before 29.2%; after 20.3%). The trends in the sedentary lifestyle style (number of those who are not practicing any physical activity) jumped from 1512 to 2269 which was a significant increase (P < 0.001). Also, the number of people watching TV increased from < 1 h to > 2 h for 24% of the participants during confinement compared with 10% only in the usual conditions. Similarly, the time spent on social media has increased from < 1 h to > 2 h for 57.1%, which was significant (P < 0.001) (Table 2).

**Table 1:** Lifestyle changes in before and during COVID-19

Items	Mean	SD	95% CI		t	Sig. (2-tailed)
			Lower	Upper		
How many times do you smoke per day before?	1.30	0.75	− 0.02	0.00	− 1.04	0.30
How many times do you smoke per day during	1.30	0.77				
How many hours do you sleep per day before?	1.53	0.59	− 0.33	− 0.29	− 28.81	0.00**
How many hours do you sleep per day during?	1.84	0.75				
How many times do you practice physical activity per week before?	2.22	1.43	0.02	0.10	3.09	0.00**
How many times do you practice physical activity per week during?	2.16	1.43				
How many minutes do you spend per each exercise before?	1.61	0.89	0.01	0.07	2.97	0.00**
How many minutes do you spend per each exercise during	1.56	0.86				
Before confinement, what were your physical activities	1.56	2.21	− 0.37	− 0.24	− 8.91	0.00**
During confinement, what are your physical activities?	1.86	2.41				
How many hours do you spend watching TV per day before?	2.27	1.41	− 0.69	− 0.63	− 43.56	0.00**
How many hours do you spend watching TV per day during?	2.93	1.58				
How many hours do you spend on social media per day before?	3.90	1.12	− 0.45	− 0.41	− 39.94	0.00**
How many hours do you spend on social media per day during?	4.33	0.95				
How many hours do you spend on the internet to (study/work) per day before?	3.65	1.35	− 0.34	− 0.29	− 26.36	0.00**
How many hours do you spend on the internet to (study/work) per day during	3.97	1.31				
How many hours do you spend with your family before?	3.67	1.33	− 0.57	− 0.52	− 37.91	0.00**
How many hours do you spend with your family during	4.21	1.14				

**Significant differences between before and during for each item

**Table 2:** Changes in time spent on TV, social media, internet, and with family before and during COVID-19

	TV		Social media		Internet (study/work)		Family	
	pre-COVID-19	during COVID-19	pre-COVID-19	during COVID-19	pre-COVID-19	during COVID-19	pre-COVID-19	during COVID-19
None	2620 (44.4)	1755 (29.8)	176 (3)	103 (1.7)	547 (9.3)	462 (7.8)	554 (9.4)	294 (5)
< 1 h/day	1090 (18.5)	852 (14.5)	606 (10.3)	255 (4.3)	822 (13.9)	589 (10)	730 (12.4)	323 (5.5)
1 h/day	794 (13.5)	724 (12.3)	1060 (18)	548 (9.3)	974 (16.5)	616 (10.4)	973 (16.5)	540 (9.2)
2 h/day	776 (13.2)	1132 (19.2)	1808 (30.7)	1624 (27.5)	1292 (21.9)	1206 (20.5)	1497 (25.4)	1391 (23.6)
> 2 h/day	616 (10.4)	1433 (24.3)	2246 (38.1)	3366 (57.1)	2261 (38.3)	3023 (51.3)	2142 (36.3)	3348 (56.8)

Data presented as n (%)

Concerning smoking, there were no significant changes in the number of smoking per day. (Table 3).

**Table 3:** Smoking habit before and during COVID-19

	Smoking pre-COVID-19	Smoking during COVID-19
Never	4910 (83.3)	4928 (83.6)
< 5 cigarettes/day	479 (8.1)	439 (7.4)
5–10 cigarettes/day	234 (4)	228 (3.9)
> 10 cigarettes/day	273 (4.6)	301 (5.1)

Data presented as n (%)

### Eating habits changes during the COVID-19 emergency

Regarding eating habits during the confinement, 2665 (44.3%) reported that their eating habits did not change. Besides, 1861 (30.9%) reported an improvement in their eating habits compared with 1492 (24.8%) reported worsening of their eating habits. Consumption of fruits, vegetables, poultry and meat, snacks, sugars, water intake increased significantly (Table 4).

**Table 4:** Daily eating habits before and after COVID-19

Items	Mean	SD	95% CI		t	Sig (2-tailed)
			Lower	Upper		
How many times do you eat fruits and vegetables per week before	2.67	1.15	− 0.232	− 0.185	− 17.164	0.00**
How many times do you eat fruits and vegetables per week during	2.88	1.13				
How many times do you eat carbohydrates per day before	3.11	1.08	− 0.182	− 0.139	− 14.614	0.00**
How many times do you eat carbohydrates per day during	3.27	1.09				
How many times do you eat meats and poultry per week before	3.66	1.17	− 0.089	− 0.053	− 7.919	0.00**
How many times do you eat meats and poultry per week during	3.73	1.17				
How many times do you eat seafood per week before	2.00	0.92	0.002	0.036	2.168	0.00**
How many times do you eat seafood per week during	1.98	0.96				
How many liters (L) of water do you drink per day before	2.60	1.02	− 0.235	− 0.194	− 20.642	0.00**
How many liters (L) of water do you drink per day during	2.82	1.02				
How many dairy products do you consumer per day before	2.41	0.89	− 0.132	− 0.099	− 13.348	0.00**
How many dairy products do you consume per day during	2.52	0.91				
How many eggs do you consume per week before?	2.77	1.11	− 0.154	− 0.114	− 13.185	0.00**
How many eggs do you consume per week during?	2.90	1.10				
How many teaspoons of sugar do you consume per day before	2.75	1.11	− 0.062	− 0.024	− 4.514	0.00**
How many teaspoons of sugar do you consume per day during	2.80	1.12				
How many snacks do you consume per week before?	2.58	1.11	− 0.066	− 0.015	− 3.145	0.002**
How many snacks do you consume per week during?	2.62	1.15				
How many times do you eat fast food per week before?	2.37	1.07	0.464	0.523	32.598	0.00**
How many times do you eat fast food per week during?	1.88	1.02				
What are the dietary supplements do you consume per day before?	3.59	1.93	0.394	0.478	20.336	0.00**
What are the dietary supplements do you consume per day during	3.16	2.12				

Besides, 41.6% of the participants declared to eat more vegetables and fruits, and the differences were significant (P < 0.01). Also, the same behavior has been noticed for the carbohydrates and meat and poultry with a percentage of 16.9% and 32.6% respectively (P < 0.01) (Table 5). On the other side, seafood consumption was significantly decreased during the lockout from 46.6% reported to eat only once a week to 41.1% (P < 0.01). Dairy products, eggs, and sugar consumption have increased, in addition to the number of snacks, and these changes were significant (Tables 6 and 7). However, fast-food consumption decreased significantly, 48.8% of the study population reported the elimination of fast food from the diet compared with 25.4% before the lockdown (Table 7). There were no differences between males and females in fast food consumption before the lockdown (r^2^ = 0.55; P = 0.49), however, during the lockdown changes were significant, females tend to decrease fast-food consumption higher than males (r^2^ = 56; P < 0.01).

**Table 5:** Frequencies of food consumption before and during COVID-19

	Fruits and vegetables		Carbohydrates		Meat and poultry		Seafood	
	Pre-COVID-19	During COVID-19	Pre-COVID-19	During COVID-19	Pre-COVID-19	During COVID-19	Pre-COVID-19	During COVID-19
None			253 (4.3)	182 (3.1)	248 (4.2)	246 (4.2)	1837 (31.2)	2082 (35.3)
Once	1242 (21.1)	977 (16.6)	1547 (26.2)	1320 (22.4)	851 (14.4)	752 (12.8)	2750 (46.6)	2423 (41.1)
Twice	1392 (23.6)	1132 (19.2)	2116 (35.9)	2025 (34.3)	1291 (21.9)	1220 (20.7)	878 (14.9)	929 (15.8)
Thrice	1244 (21.1)	1334 (22.6)	1177 (20)	1371 (23.3)	1725 (29.3)	1755 (29.8)	321 (5.4)	334 (5.7)
More than thrice	2018 (34.2)	2453 (41.6)	803 (13.6)	998 (16.9)	1781 (30.2)	1923 (32.6)	110 (1.9)	128 (2.2)

Data presented as n (%)

**Table 6:** Frequencies of water, egg, and sugar consumption before and during COVID-19

		Pre-COVID-19	During COVID-19
Water			
a)	< 1 L/day	630 (10.7)	901 (15.3)
	1 L/day	1450 (24.6)	1725 (29.3)
	2 L/day	2469 (41.9)	2282 (38.7)
	3 L/day	1014 (17.2)	746 (12.7)
	> 3 L/day	333 (5.6)	242 (4.1)
Egg			
b)	None	1034 (17.5)	926 (15.7)
	1 egg	1301 (22.1)	1083 (18.4)
	2 eggs	1506 (25.5)	1486 (25.2)
	≥ 3 eggs	2055 (34.9)	2401 (40.7)
Sugar			
c)	None	1115 (18.9)	1119 (19)
	1 teaspoon	1037 (17.6)	1146 (19.4)
	2 teaspoon	1666 (28.3)	1689 (28.6)
	≥ 3 teaspoon	2078 (35.2)	1942 (32.9)

Data presented as n (%)

**Table 7:** Frequencies of dairy products, snacks, and fast food consumption before and during COVID-19

	Dairy product		Snacks		Fast food	
	Pre-COVID-19	During COVID-19	Pre-COVID-19	During COVID-19	Pre-COVID-19	During COVID-19
None	768 (13)	685 (11.6)	1244 (21.1)	1320 (22.4)	1499 (25.4)	2879 (48.8)
Once	2753 (46.7)	2434 (41.3)	1624 (27.5)	1446 (24.5)	1888 (32)	1499 (25.4)
Twice	1532 (26)	1748 (29.6)	1370 (23.2)	1261 (21.4)	1305 (22.1)	884 (15)
Thrice and more	843 (14.3)	1029 (17.5)	1658 (28.1)	1869 (31.7)	1204 (20.4)	634 (10.8)

Data presented as n (%)

### Impact of COVID-19 infection on lifestyle changes

According to the information collected, 14% of the respondents reported being infected with COVID-19 or living with an infected household. This direct/indirect exposure to COVID-19 infection was associated with an increased consumption of carbohydrates (OR = 1.09; 95% CI = 1.02–1.17; P = 0.01), egg (OR = 1.08; 95% CI = 1.02–1.16; P = 0.01), sugar (OR = 1.09; 95% CI = 1.02–1.16; P = 0.02), meat, and poultry consumption (OR = 1.13; 95% CI = 1.06–1.20; P < 0.01). There was also associated increase in hours spent on TV (OR = 1.07; 95% CI = 1.02–1.12; P < 0.01) and Social media (OR = 1.09; 95% CI = 1.01–1.18; P = 0.03). However, among those participants, a reduction in utilizing vitamin C (OR = 0.26; 95% CI = 0.15–0.44; P < 0.01), vitamin D (OR = 0.50; 95% CI = 0.28–0.87; P = 0.01), multivitamins (OR = 0.11; 95% CI = 0.06–0.20; P < 0.01) was observed. A similar significant reduction in sleeping hours was identified among those exposed to COVID-19 infection (OR = 0.85; 95% CI = 0.77–0.94; P < 0.01). The binary logistic of the significant variables among COVID-19-exposed participants are shown in Table 8.

**Table 8:** Binary logistic of the significant variables during COVID-19 infection

	Intercept	Standard error	Wald chi-square	Degrees of freedom	Sig	Exp(B)	95% CI for EXP(B)	
							Lower	Upper
Dietary habits								
Daily carbohydrates consumption	0.088	0.035	6.379	1	0.012	1.092	1.020	1.169
Weekly meats and poultry consumption	0.121	0.032	14.791	1	0.000	1.129	1.061	1.201
Weekly eggs consumption	0.084	0.034	6.282	1	0.012	1.088	1.019	1.162
Daily usage of teaspoons	0.082	0.034	5.929	1	0.015	1.085	1.016	1.160
Supplements								
Vitamin C	− 1.365	0.277	24.282	1	0.000	0.255	0.148	0.439
Vitamin D	− 0.700	0.286	6.004	1	0.014	0.497	0.284	0.869
Multivitamins	− 2.228	0.324	47.393	1	0.000	0.108	0.057	0.203
None	− 0.924	0.288	10.279	1	0.001	0.397	0.226	0.698
Lifestyle habits								
Daily sleeping hours	− 0.159	0.051	9.846	1	0.002	0.853	0.772	0.942
Daily watching TV hours	0.068	0.024	8.022	1	0.005	1.070	1.021	1.121
Daily hours spent on social media	0.086	0.038	5.032	1	0.025	1.090	1.011	1.175

## Discussion

This study describes the impact of COVID-19 on lifestyle behaviors in MENA region. Our findings show that sedentary lifestyle activities increased, such as spending more hours on TV, social media, and increases the consumption of meat, poultry and vitamins. As a result, 21.3% have reported an increase in weight during confinement. Besides, we estimated a 42% increase in people who stopped practicing sport during the confinement. According to Woods et al., physical inactivity due to sustained quarantine and social distancing can downregulate the ability of the body to resist viral infection and increase the risk of damage to the immune, respiratory, cardiovascular, musculoskeletal systems, and the brain [17]. Our results might help to develop appropriate lifestyle behaviors for the populations in MENA region. Sedentary habits are associated with body fat and appetite dysregulation [18]. Thus, it is vital to maintain a correct nutrition status, particularly in COVID-19 time, when the first defense line is the immune system. According to the Centers for Disease Control and Prevention (CDC), individuals with obesity (BMI ≥ 30 kg/m^2^) are at increased risk of COVID-19 severe illness [19]. Obesity is known as a proinflammatory condition; both hypertrophied adipocytes and adipose tissue-resident immune cells are contributing to increasing circulating pro-inflammatory cytokines levels [20]. Besides, respiratory physiology is altered in obesity since it decreases expiratory reserve volume and functional residual capacity. While lying horizontally in the supine position, pulmonary function is also being compromised due to the presence of high abdominal fat which decreases diaphragmatic excursion, in such a state, ventilation becomes more problematic [21]. Moreover, in COVID-19, it is crucial to identify the inflammatory condition because it is one of the essential factors that is used in the determination of lung disease severity which results in hypercytokinemia associated with acute respiratory distress syndrome and multiple organ failure [10]. Therefore, in obese people, the situation might be exacerbated as a result of having higher proinflammatory cytokine levels compared with normal-weight people [10]. Besides, it is essential to keep a healthy diet because the entire cytokines gene expression levels are included by the food [22] and can modulate the inflammation process and oxidative stress [22].

In this study, some people shifted their outdoor activities to indoor-only activities. Outdoor activities such as running, walking, football, swimming have decreased by 23%, 30%, 57%, and 51%, respectively. On the other side, confinement gave people time to practice their hobbies, such as cooking, house duties, and drawing. Time spent with families has changed among individuals, married people declared that they spent much time with their families during the confinement. According to Arafat et al., there was an increase in couples' sexual activity as a result of higher reassurance and intimacy or simply because of spending more time together [15]. Muise et al. al show that spending more time with the family increases the emotional bonding among family members [23]. These activities can change the moods of the individuals, which may affect their life in many ways. The impact of pessimistic emotions has shown to be associated with overeating represented as “emotional eating” [24]. The adverse effect of self-isolation support people looking for reward and gratification physiologically linked to food consumption [25]. Moreover, in the above-mentioned status, boredom feelings could be an expected result which is usually associated with overeating as a method to escape monotony [26]. Similarly, adverse events have been reported to be associated with disordered eating habits [27].

**Fig. 1:**
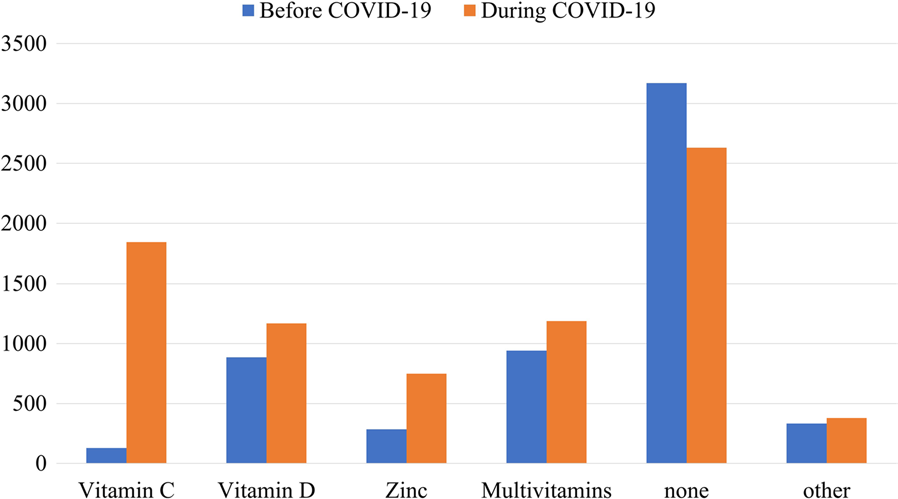
Dietary supplementation before and during COVID-19 emergency

It can be challenging during the quarantine time to maintain healthy food and regular physical activity. For instance, vegetables, fruits, and fish consumption can decrease in favor of canned food, fast meals, snacks, and ready-to-eat foods, which have a higher amount of sugars, fats, and salts. Besides, there is an increased risk to develop dysfunctional eating behaviors as a result of emotional and psychological responses to the COVID-19 outbreak [11, 28]. Regarding eating habits in this study, 30.9% reported an improvement in their eating habits compared with 24.8% reported a worsening of such habits, and the rest remain the same with no changes. Dietary supplement consumption was also deemed significant, with a rate of 1328.7% for vitamin C, 31.8% for vitamin D, 163% for Zinc, and 26.2% for multivitamins. This rate is associated with various publications that recommended vitamin C during COVID-19 [29, 30], vitamin D [31], and Zinc [32] (Fig. 1). The regular consumption of these vitamins is not common in the MENA region since they depend on their intake of food, particularly for vitamin C and Zinc. It is known that the sun is essential for vitamin D synthesis, which, when deficient, is associated with various diseases [33]. However, it was reported by Chakhtoura et al. that the majority of the MENA region population is vitamin D deficient [34]. Overall, there was an increase in food consumption during the confinement. However, consumption of seafood showed a significant decrease. This is due to government restrictions on several occupations, including fishers. Also, positive aspects of improving health habits were associated with decreasing the consumption of fast food, and that was positively associated with increasing cooking as a hobby. Since there are several studies that investigated the impact of COVID-19 on the lifestyle, Table 9 represents a comparison between the results of the current research and previous studies conducted by Constant et al. [35], Di Renzo et al. [36], and Ammar et al. [37].

**Table 9:** Studies investigated the impact of COVID-19 on lifestyle

Confinement dimension	Current study (n = 5896, MENA)	Constant et al. [35] (n = 4005, France)	Di Renzo et al. [36] (n = 3533, Italy)	Ammar et al. [37] (n = 1047, International)
Physical activity	Decline in number of weekly exercises before and during confinement (2.22 ± 1.43 vs. 2.16 ± 1.43; P < 0.001)^¶^. 38.4% of the participants stopped practicing any physical activities (P < 0.001).	Decline in regular exercise and walking in 45% and 60% of the participants, respectively.	Increase in the weekly training frequency ≥ 5 times before and during confinement in 6.1% vs. 16.4% of the participants. (McNemar value = 259.529; P < 0.001).	Decline in number of weekly exercises before and during confinement (5.04 ± 2.51 vs. 3.83 ± 2.82; P < 0.001)^¶^.
Smoking & alcohol	No significant changes in smoking habits. Alcohol consumption was not measured.	Slight increase in tobacco in regular smokers. Decline in alcohol consumption in regular drinkers.	Decline in smoking habits (McNemar value = 101.484, P < 0.001). Decline in alcohol consumption.	Smoking was not measured. Decline in alcohol consumption (*t* = − 12.16, *P* < 0.001, *d* = 0.58).
Eating behaviors	Improvement of eating habits in 30.9% of the participants. Worsening of eating habits in 24.8% of the participants. Decline in fast-food consumption in 48.8% of the participants (P < 0.001). Increase in vegetables and fruits consumption in 41.6% of the participants (P < 0.001) Before lockdown: no difference was found for gender regarding eating behaviors (r^2^ = 0.55; P = 0.49). During confinement: females tend to decrease fast-food consumption higher than males (r^2^ = 56; P < 0.01).	Improvement of eating habits in less than 40% of the participants. This improvement was associated with living with more than two persons. Worsening of eating habits has been noticed in elder participants with ages ≥ 40 years. Decline in snack consumption in 18% of the participants. Increase in snack consumption in 24% of the participants.	No changes in the frequency of daily in 57.8% of the participants. Changing in the main- mealtime or introduction of a break between meals was reported by 23.5% of the participants. Skipping the meals was reported by 17.5% of the participants. Increase in purchasing vegetables and fruits from farmers and organic grocery stores was reported by 15% of the participants. Decline in fast-food consumption in 29.8% of the participants (r^2^ = 9.560, P = 0.002). No difference was found for gender regarding eating behaviors.	Consuming unhealthy food was significantly higher during home confinement (*t* = − 3.46, *P* < 0.001, *d* = 0.14).
Weight gain	Increase of the weight in 21.3% of the participants.	NA	Weight gain was inversely and positively associated with the consumption of healthy food or fast-food, respectively (OR = 0.805, P = 0.002; OR = 3.122, P < 0.001)	NA
Dietary supplementation	Significant increase in dietary supplement consumption, with a rate of 1328.7% for vitamin C, 31.8% for vitamin D, 163% for Zinc, and 26.2% for multivitamins	NA		

Data presented as mean ± SD

One of the main limitations of a web survey is self-reported data, which could be prone to bias and misreporting, which applies to our survey as well. Further, representation of the population is difficult as participants were recruited using snowball sampling leading to selection bias. A strength of this study is the collection of large data from the MENA region during the critical time, which can suggest public health policies for people in the region.

## Conclusion

Our results show that sedentary lifestyle increased as seen by reduced physical activities and spending more time on social media and television. Sleep time reduced and body weight increased in a large number of people during the pandemic. Although fast-food consumption significantly decreased after the lockdown, there was a significant increase in the rate of dietary supplement consumption, including vitamin C, vitamin D, and Zinc. Exposure to COVID-19 by direct infection or through an infected household member is a significant predictor of amplifying these changes. Public health interventions should be developed to reduce these hazardous effects and avoid the emergence of a deadlier pandemic.

## EARG COLLABORATORS

Akram Ramadan Farag Elburki, Almajdoub Ali Mohammed Ali, Moufiq Abdulrasul Hasan, Emadaldin Awad Amgrab ALI, Mahmoud N. Gumma Mohamed, Mareb H. Ahmed, Ayat K. Taher, Luma Saad Abdalbaqi, Nuha Hadi Jasim, Ibrahim Adel Aziz, Amna Babiker Dafaallah, Sirine Ben Dalla, Omar Mastouri, Chams Zarrad, Asmaa E. Abdelslam, Ahmed Taha A. Sayed, Kyrillos Wassim, Mahmoud Shaban, Tarek Mohamed Fayez, Mohammed Nasser, Zahraa Radhi, Zahraa Alkuwaiti, Amina Alsaffar, Asmaa M. Elaidy, Majeda Hammoud, Sabri M. Hammoud, Ali Saheb Alturki, Tasneem Jarkhi, Rama Hamza, Nourelhuda Mahmoud Issa, Reham Ahmad Kaakeh, Omar Alfatohi Aljondi, Ahmed Ali Ahmed, Mona H. Ibrahim, Islam Samy, Fatima Abdulrahman, Walaa Sabry Fouad, Normala Shahin, Saif Salman, Sumaia Ghunaim, Sarah Elayyan, Farah Ahmed Alkhaled, Deena Elsori, Samy Nael Altayeb, Elias Edward Lahham, Afnan W.M. Jobran, Asil Nael Salman, Aroub Imad Abdelhaq, Ola Hassan Akkawi, Ali Hassan Salah Al-Hadi, Sadeq Nagi Rashed Gozelan, Hatem Sultan Ahmed Qasim, Abdullah Ahmed Areqi, Azal Abduulah Abdurab Thabet, Boutheyna Drid, Sara Menzer, Melissa Hamdani, Zighed Alya, Najlae Adadi, Zakaria El Hamri, Hinde El Mouhi.

## Supplementary Information

**Additional file 1. **Questionnaire.

**Additional file 2.** Tables S1, S2, S3 and S4.

**Additional file 3.** Survey Development.

## Data Availability

All data generated or analysed during this study are included in this published article [Additional file 1: Questionnaire; Additional file 2: Tables S1, S2, S3 and S4; Additional file 3: Survey Development]. Original dataset/raw data are available from the corresponding author on reasonable request.

## References

[1] Yu ES, Lange JJ, Broor A, Kutty K (2019). Acute pancreatitis masquerading as inferior wall myocardial infarction: a review. Case Rep Gastroenterol.

[2] COVID-19 crisis response in MENA countries. OECD 2020.

[3] Cucinotta D, Vanelli M (2020). WHO Declares COVID-19 a Pandemic. Acta Bio-Medica Atenei Parmensis.

[4] Sheth J (2020). Impact of Covid-19 on Consumer Behavior: Will the Old Habits Return or Die?. J Bus Res.

[5] Nicola M, Alsafi Z, Sohrabi C, Kerwan A, Al-Jabir A, Iosifidis C, Agha M, Agha R (2020). The socio-economic implications of the coronavirus and COVID-19 pandemic: a review. Int J Surg.

[6] Gray C, Gibbons R, Larouche R, Sandseter EBH, Bienenstock A, Brussoni M, Chabot G, Herrington S, Janssen I, Pickett W (2015). What is the relationship between outdoor time and physical activity, sedentary behaviour, and physical fitness in children? a systematic review. Int J Environ Res Public Health.

[7] Moynihan AB, Tilburg WAP, Igou ER, Wisman A, Donnelly AE, Mulcaire JB (2015). Eaten up by boredom: consuming food to escape awareness of the bored self. Front Psychol.

[8] Sinha R, Gu P, Hart R, Guarnaccia JB (2019). Food craving, cortisol and ghrelin responses in modeling highly palatable snack intake in the laboratory. Physiol Behav.

[9] Rodríguez-Martín BC, Meule A (2015). Food craving: new contributions on its assessment, moderators, and consequences. Front Psychol.

[10] Muscogiuri G, Pugliese G, Barrea L, Savastano S, Colao A (2020). Commentary: Obesity: The “Achilles heel” for COVID-19?. Metab Clin Exp.

[11] Wu C, Chen X, Cai Y, Ja X, Zhou X, Xu S, Huang H, Zhang L, Zhou X (2020). Risk factors associated with acute respiratory distress syndrome and death in patients with coronavirus disease 2019 Pneumonia in Wuhan, China. JAMA Intern Med.

[12] Huang Y, Zhao N (2020). Generalized anxiety disorder, depressive symptoms and sleep quality during COVID-19 outbreak in China: a web-based cross-sectional survey. Psychiatry Res.

[13] Meyer KA, Wall MM, Larson NI, Laska MN, Neumark-Sztainer D (2012). Sleep Duration and BMI in a Sample of Young Adults. Obesity.

[14] Berlin I, Thomas D, Le Faou A-L, Cornuz J (2020). COVID-19 and Smoking. Nicotine Tob Res.

[15] Arafat SMY, Alradie-Mohamed A, Kar SK, Sharma P, Kabir R (2020). Does COVID-19 pandemic affect sexual behaviour? A cross-sectional, cross-national online survey. Psychiatry Res.

[16] Steel RGD, Torrie JH, Dickey DA (1996). Principles and Procedures of Statistics: A Biometrical Approach.

[17] Woods JA, Hutchinson NT, Powers SK, Roberts WO, Gomez-Cabrera MC, Radak Z, Berkes I, Boros A, Boldogh I, Leeuwenburgh C (2020). The COVID-19 pandemic and physical activity. Sports Med Health Sci.

[18] Panahi S, Tremblay A (2018). Sedentariness and health: is sedentary behavior more than just physical inactivity?. Front Public Health.

[19] CDC. Coronavirus Disease 2019 (COVID-19). Centers for Disease Control and Prevention 2020.

[20] Hauner H (2005). Secretory factors from human adipose tissue and their functional role. Proc Nutr Soc.

[21] Engin AB, Engin ED, Engin A (2020). Two important controversial risk factors in SARS-CoV-2 infection: obesity and smoking. Environ Toxicol Pharmacol.

[22] Di Renzo L, Gualtieri P, Romano L, Marrone G, Noce A, Pujia A, Perrone MA, Aiello V (2019). Colica C.

[23] Muise A, Schimmack U, Impett EA (2016). Sexual frequency predicts greater well-being, but more is not always better. Soc Psychol Person Sci.

[24] Annesi JJ (2020). Psychosocial correlates of emotional eating and their interrelations: implications for obesity treatment research and development. J Primary Prevent.

[25] Volkow ND, Wang G-J, Baler RD (2011). Reward, dopamine and the control of food intake: implications for obesity. Trends Cogn Sci.

[26] Crockett AC, Myhre SK, Rokke PD (2015). Boredom proneness and emotion regulation predict emotional eating. J Health Psychol.

[27] Smyth JM, Heron KE, Wonderlich SA, Crosby RD, Thompson KM (2008). The influence of reported trauma and adverse events on eating disturbance in young adults. Int J Eat Disord.

[28] Montemurro N (2020). The emotional impact of COVID-19: From medical staff to common people. Brain Behav Immun.

[29] Kim SB, Yeom JS (2020). Reply: Vitamin C as a Possible therapy for COVID-19. Infect Chemother.

[30] Simonson W (2020). Vitamin C and coronavirus. Geriatric Nursing (New York, Ny).

[31] Martineau AR, Forouhi NG (2020). Vitamin D for COVID-19: a case to answer?. Lancet Diabetes Endocrinol.

[32] Wessels I, Rolles B, Rink L (2020). The potential impact of zinc supplementation on COVID-19 pathogenesis. Front Immunol.

[33] Abouzid M, Karazniewicz-Lada M, Glowka F (2018). Genetic determinants of vitamin D-related disorders; focus on vitamin D receptor. Curr Drug Metab.

[34] Chakhtoura M, Rahme M, Chamoun N, El-Hajj Fuleihan G (2018). Vitamin D in the Middle East and North Africa. Bone Reports.

[35] Constant A, Conserve DF, Gallopel-Morvan K, Raude J (2020). Socio-cognitive factors associated with lifestyle changes in response to the COVID-19 epidemic in the general population: results from a cross-sectional study in France. Front Psychol.

[36] Di Renzo L, Gualtieri P, Pivari F, Soldati L, Attina A, Cinelli G (2020). Eating habits and lifestyle changes during COVID-19 lockdown: an Italian survey. J Transl Med.

[37] Ammar A, Brach M, Trabelsi K, Chtourou H, Boukhris O, Masmoudi L (2020). Effects of COVID-19 home confinement on eating behaviour and physical activity: results of the ECLB-COVID19 International Online Survey. Nutrients.

