# Supplementary material for: Influence of COVID-19 on lifestyle behaviors in the Middle East and North Africa Region: a survey of 5896 individuals
Source: J Transl Med. 2021 Mar 30;19:129. doi: 10.1186/s12967-021-02767-9 (PMC8008335; doi:10.1186/s12967-021-02767-9)
Supplement: Supplementary file 1 — Additional file 1. Questionnaire. [file 12967_2021_2767_MOESM1_ESM.docx]

**personal data**

1. Age

- 18-23
- 24-30
- 31-40
- 41-60
- >60

1. Sex

- Male
- Female

1. Resident country

- Egypt
- Jordan
- United Arab Emirates
- Kuwait
- Bahrain
- Saudi Arabia
- Oman
- Qatar
- Yemen
- Syria
- Lebanon
- Palestine
- Algeria
- Morocco
- Libya
- Tunisia
- Iraq
- Sudan

1. Height (cm)
2. Weight (kg)
3. Social status

- Single
- Married
- Divorced/Widower

1. Living area

- City
- Countryside
- Costal
- Desert

1. Occupation

- Regular employee
- Shift employee
- Freelancer
- Do not work
- Student
- Left work because of COVID-19
- Medical field

1. Education level

- Basic
- Secondary
- Higher education
- Postgraduate studies
- Other

1. Did you or any related household have the coronavirus infection?

- Yes
- No

1. Do you have chronic diseases?

- No
- Cardiovascular disease
- Diabetes
- Cancer
- Kidney diseases
- Hypertension
- High cholesterol
- Requires medical attention
- Other

**Daily eating habits**

1. Did you change your eating habits during confinement?

- No
- Yes, for better
- Yes, for worse

1. Did your weight change from before confinement?

- No
- Yes, increased
- Yes, decreased

1. How many times do you eat fruits and vegetables per week?

| Before confinement | Once | Twice | thrice | More than thrice |
| --- | --- | --- | --- | --- |
| After confinement | Once | Twice | thrice | More than thrice |

1. How many times do you eat carbohydrates per day?

| Before confinement | None | Once | Twice | thrice | More than thrice |
| --- | --- | --- | --- | --- | --- |
| After confinement | None | Once | Twice | thrice | More than thrice |

1. How many times do you eat meats and poultry per week?

| Before confinement | None | Once | Twice | thrice | More than thrice |
| --- | --- | --- | --- | --- | --- |
| After confinement | None | Once | Twice | thrice | More than thrice |

1. How many times do you eat seafood per week?

| Before confinement | None | Once | Twice | thrice | More than thrice |
| --- | --- | --- | --- | --- | --- |
| After confinement | None | Once | Twice | thrice | More than thrice |

1. How many litres (L) of water do you drink per day?

| Before confinement | < 1 L | 1 L | 2 L | 3 L | > 3 L |
| --- | --- | --- | --- | --- | --- |
| After confinement | < 1 L | 1 L | 2 L | 3 L | > 3 L |

1. How many dairy products do you consume per day?

| Before confinement | None | Once | Twice | Thrice and more |
| --- | --- | --- | --- | --- |
| After confinement | None | Once | Twice | Thrice and more |

1. How many eggs do you consume per week?

| Before confinement | None | 1 | 2 | ≥3 |
| --- | --- | --- | --- | --- |
| After confinement | None | 1 | 2 | ≥3 |

1. How many teaspoons of sugar do you consume per day?

| Before confinement | None | 1 | 2 | ≥3 |
| --- | --- | --- | --- | --- |
| After confinement | None | 1 | 2 | ≥3 |

1. How many snacks do you consume per week?

| Before confinement | None | 1 | 2 | ≥3 |
| --- | --- | --- | --- | --- |
| After confinement | None | 1 | 2 | ≥3 |

1. How many times do you eat fast food per week?

| Before confinement | None | 1 | 2 | ≥3 |
| --- | --- | --- | --- | --- |
| After confinement | None | 1 | 2 | ≥3 |

1. What are drinks do you consume per day?

| Before confinement | Caffeinated | Traditional | Juice | Other |
| --- | --- | --- | --- | --- |
| After confinement | Caffeinated | Traditional | Juice | Other |

1. How many cups of drinks do you consume per day?

| Before confinement | None, only water | 1 | 2 | ≥3 |
| --- | --- | --- | --- | --- |
| After confinement | None, only water | 1 | 2 | ≥3 |

1. What are the dietary supplements do you consume per day?

| Before confinement | Vitamin C | Vitamin D | Zinc | Multivitamins | None | Other |
| --- | --- | --- | --- | --- | --- | --- |
| After confinement | Vitamin C | Vitamin D | Zinc | Multivitamins | None | Other |

**lifestyle**

1. How many times do you smoke per day?

| Before confinement | Never | <5 | 5-10 | >10 |
| --- | --- | --- | --- | --- |
| After confinement | Never | <5 | 5-10 | >10 |

1. How many hours do you sleep per day?

| Before confinement | <7 | 7-10 | 10-12 | >12 |
| --- | --- | --- | --- | --- |
| After confinement | <7 | 7-10 | 10-12 | >12 |

1. How many times do you practise physical activity per week?

| Before confinement | None | 1 | 2 | 3 | 4-5 | >5 |
| --- | --- | --- | --- | --- | --- | --- |
| After confinement | None | 1 | 2 | 3 | 4-5 | >5 |

1. How many minutes do you spend per each exercise?

| Before confinement | 30 | 60 | 90 | 120 | >120 |
| --- | --- | --- | --- | --- | --- |
| After confinement | 30 | 60 | 90 | 120 | >120 |

1. Before confinement, what were your physical activities

- None
- Walking
- Running
- Swimming
- Yoga
- Indoor activities
- Football
- Basketball
- Volleyball
- Cardio
- Weightlifting
- Other

1. During confinement, what are your physical activities?

- None
- Walking
- Running
- Swimming
- Yoga
- Indoor activities
- Football
- Basketball
- Volleyball
- Cardio
- Weightlifting
- Other

1. How many hours do you spend watching TV per day?

| Before confinement | None | <1 | 1 | 2 | >2 |
| --- | --- | --- | --- | --- | --- |
| After confinement | None | <1 | 1 | 2 | >2 |

1. How many hours do you spend on social media per day?

| Before confinement | None | <1 | 1 | 2 | >2 |
| --- | --- | --- | --- | --- | --- |
| After confinement | None | <1 | 1 | 2 | >2 |

1. How many hours do you spend on the internet to (study/work) per day?

| Before confinement | None | <1 | 1 | 2 | >2 |
| --- | --- | --- | --- | --- | --- |
| After confinement | None | <1 | 1 | 2 | >2 |

1. How many hours do you spend with your family?

| Before confinement | None | <1 | 1 | 2 | >2 |
| --- | --- | --- | --- | --- | --- |
| After confinement | None | <1 | 1 | 2 | >2 |
