# Supplementary material for: Influence of COVID-19 on lifestyle behaviors in the Middle East and North Africa Region: a survey of 5896 individuals
Source: J Transl Med. 2021 Mar 30;19:129. doi: 10.1186/s12967-021-02767-9 (PMC8008335; doi:10.1186/s12967-021-02767-9)
Supplement: Supplementary file 2 — Additional file 2. Tables S1, S2, S3 and S4. [file 12967_2021_2767_MOESM2_ESM.docx]

| **Table S1. Participants’ general characteristics and anthropometrics** | | | | | | | | | | | |
| --- | --- | --- | --- | --- | --- | --- | --- | --- | --- | --- | --- |
|  | **countries** | **n %** | **Age** | | | | | **Sex** | | **Height (cm)** | **Weight (kg)** |
|  |  |  | **18-23** | **24-30** | **31-40** | **41-60** | **> 60** | **M** | **F** |  |  |
| **1** | **Egypt** | 469.0 | 326.0 | 81.0 | 28.0 | 32.0 | 2.0 | 180.0 | 289.0 | 166.26 | 72.36 |
|  |  |  | 69.5 | 17.3 | 6.0 | 6.8 | 0.4 | 38.4 | 61.6 |  |  |
| **2** | **Jordan** | 414.0 | 273.0 | 57.0 | 38.0 | 35.0 | 11.0 | 113.0 | 301.0 | 167.12 | 67.16 |
|  |  |  | 65.9 | 13.8 | 9.2 | 8.5 | 2.7 | 27.3 | 72.7 |  |  |
| **3** | **United Arab Emirates** | 407.0 | 81.0 | 87.0 | 125.0 | 107.0 | 7.0 | 119.0 | 288.0 | 161.27 | 74.01 |
|  |  |  | 19.9 | 21.4 | 30.7 | 26.3 | 1.7 | 29.2 | 70.8 |  |  |
| **4** | **Kuwait** | 430.0 | 32.0 | 54.0 | 125.0 | 177.0 | 42.0 | 126.0 | 304.0 | 166.43 | 76.89 |
|  |  |  | 7.4 | 12.6 | 29.1 | 41.2 | 9.8 | 29.3 | 70.7 |  |  |
| **5** | **Bahrain** | 438.0 | 198.0 | 87.0 | 88.0 | 63.0 | 2.0 | 111.0 | 327.0 | 159.50 | 69.34 |
|  |  |  | 45.2 | 19.9 | 20.1 | 14.4 | 0.5 | 25.3 | 74.7 |  |  |
| **6** | **Saudi Arabia** | 419.0 | 29.0 | 78.0 | 196.0 | 106.0 | 10.0 | 242.0 | 177.0 | 169.95 | 84.83 |
|  |  |  | 6.9 | 18.6 | 46.8 | 25.3 | 2.4 | 57.8 | 42.2 |  |  |
| **7** | **Oman** | 13.0 | 6.0 | 0.0 | 4.0 | 3.0 | 0.0 | 3.0 | 10.0 | 159.84 | 65.46 |
|  |  |  | 46.2 | 0.0 | 30.8 | 23.1 | 0.0 | 23.1 | 76.9 |  |  |
| **8** | **Qatar** | 22.0 | 7.0 | 9.0 | 3.0 | 2.0 | 1.0 | 4.0 | 18.0 | 169.25 | 60.36 |
|  |  |  | 31.8 | 40.9 | 13.6 | 9.1 | 4.5 | 18.2 | 81.8 |  |  |
| **9** | **Yemen** | 222.0 | 77.0 | 70.0 | 49.0 | 23.0 | 3.0 | 168.0 | 54.0 | 150.14 | 64.84 |
|  |  |  | 34.7 | 31.5 | 22.1 | 10.4 | 1.4 | 75.7 | 24.3 |  |  |
| **10** | **Syria** | 422.0 | 219.0 | 101.0 | 56.0 | 41.0 | 5.0 | 162.0 | 260.0 | 167.25 | 68.32 |
|  |  |  | 51.9 | 23.9 | 13.3 | 9.7 | 1.2 | 38.4 | 61.6 |  |  |
| **11** | **Palestine** | 399.0 | 313.0 | 53.0 | 12.0 | 15.0 | 6.0 | 116.0 | 283.0 | 163.17 | 63.67 |
|  |  |  | 78.4 | 13.3 | 3.0 | 3.8 | 1.5 | 29.1 | 70.9 |  |  |
| **12** | **Algeria** | 169.0 | 85.0 | 52.0 | 25.0 | 7.0 | 0.0 | 36.0 | 133.0 | 168.20 | 68.84 |
|  |  |  | 50.3 | 30.8 | 14.8 | 4.1 | 0.0 | 21.3 | 78.7 |  |  |
| **13** | **Morocco** | 146.0 | 57.0 | 43.0 | 29.0 | 16.0 | 1.0 | 66.0 | 80.0 | 165.80 | 68.74 |
|  |  |  | 39.0 | 29.5 | 19.9 | 11.0 | 0.7 | 45.2 | 54.8 |  |  |
| **14** | **Libya** | 520.0 | 72.0 | 131.0 | 181.0 | 101.0 | 35.0 | 310.0 | 210.0 | 166.35 | 80.24 |
|  |  |  | 13.8 | 25.2 | 34.8 | 19.4 | 6.7 | 59.6 | 40.4 |  |  |
| **15** | **Tunisia** | 487.0 | 291.0 | 134.0 | 47.0 | 14.0 | 1.0 | 167.0 | 320.0 | 167.59 | 68.83 |
|  |  |  | 59.8 | 27.5 | 9.7 | 2.9 | 0.2 | 34.3 | 65.7 |  |  |
| **16** | **Iraq** | 519.0 | 243.0 | 118.0 | 87.0 | 61.0 | 10.0 | 147.0 | 372.0 | 161.97 | 68.54 |
|  |  |  | 46.8 | 22.7 | 16.8 | 11.8 | 1.9 | 28.3 | 71.7 |  |  |
| **17** | **Sudan** | 509.0 | 397.0 | 73.0 | 24.0 | 14.0 | 1.0 | 168.0 | 341.0 | 166.74 | 63.75 |
|  |  |  | 78.0 | 14.3 | 4.7 | 2.8 | 0.2 | 33.0 | 67.0 |  |  |

| **Table S2. Participants’ social status and living area.** | | | | | | | | |
| --- | --- | --- | --- | --- | --- | --- | --- | --- |
|  |  | **Social status** | | | **Living area** | | | |
| **sn** | **countries** | **Single** | **Married** | **Divorced/**  **Widower** | **City** | **Countryside** | **Costal** | **Desert** |
| **1** | **Egypt** | 395.0 | 71.0 | 3.0 | 316.0 | 146.0 | 6.0 | 1.0 |
|  |  | 84.2 | 15.1 | 0.6 | 67.4 | 31.1 | 1.3 | 0.2 |
| **2** | **Jordan** | 319.0 | 94.0 | 1.0 | 373.0 | 34.0 | 4.0 | 3.0 |
|  |  | 77.1 | 22.7 | 0.2 | 90.1 | 8.2 | 1.0 | 0.7 |
| **3** | **United Arab Emirates** | 146.0 | 245.0 | 16.0 | 353.0 | 23.0 | 10.0 | 21.0 |
|  |  | 35.9 | 60.2 | 3.9 | 86.7 | 5.7 | 2.5 | 5.2 |
| **4** | **Kuwait** | 89.0 | 320.0 | 21.0 | 372.0 | 0.0 | 20.0 | 38.0 |
|  |  | 20.7 | 74.4 | 4.9 | 86.5 | 0.0 | 4.7 | 8.8 |
| **5** | **Bahrain** | 213.0 | 216.0 | 9.0 | 332.0 | 40.0 | 54.0 | 12.0 |
|  |  | 48.6 | 49.3 | 2.1 | 75.8 | 9.1 | 12.3 | 2.7 |
| **6** | **Saudi Arabia** | 84.0 | 324.0 | 11.0 | 354.0 | 12.0 | 38.0 | 15.0 |
|  |  | 20.0 | 77.3 | 2.6 | 84.5 | 2.9 | 9.1 | 3.6 |
| **7** | **Oman** | 9.0 | 14.0 | 0.0 | 11.0 | 2.0 | 0.0 | 0.0 |
|  |  | 69.2 | 107.7 | 0.0 | 84.6 | 15.4 | 0.0 | 0.0 |
| **8** | **Qatar** | 15.0 | 5.0 | 2.0 | 17.0 | 2.0 | 0.0 | 3.0 |
|  |  | 68.2 | 22.7 | 9.1 | 77.3 | 9.1 | 0.0 | 13.6 |
| **9** | **Yemen** | 122.0 | 92.0 | 8.0 | 142.0 | 47.0 | 27.0 | 6.0 |
|  |  | 55.0 | 41.4 | 3.6 | 64.0 | 21.2 | 12.2 | 2.7 |
| **10** | **Syria** | 29.0 | 118.0 | 7.0 | 390.0 | 26.0 | 6.0 | 0.0 |
|  |  | 6.9 | 28.0 | 1.7 | 92.4 | 6.2 | 1.4 | 0.0 |
| **11** | **Palestine** | 337.0 | 61.0 | 1.0 | 258.0 | 133.0 | 5.0 | 3.0 |
|  |  | 84.5 | 15.3 | 0.3 | 64.7 | 33.3 | 1.3 | 0.8 |
| **12** | **Algeria** | 143.0 | 25.0 | 1.0 | 136.0 | 12.0 | 17.0 | 4.0 |
|  |  | 84.6 | 14.8 | 0.6 | 80.5 | 7.1 | 10.1 | 2.4 |
| **13** | **Morocco** | 108.0 | 34.0 | 4.0 | 127.0 | 10.0 | 6.0 | 3.0 |
|  |  | 74.0 | 23.3 | 2.7 | 87.0 | 6.8 | 4.1 | 2.1 |
| **14** | **Libya** | 243.0 | 256.0 | 21.0 | 408.0 | 29.0 | 56.0 | 27.0 |
|  |  | 46.7 | 49.2 | 4.0 | 78.5 | 5.6 | 10.8 | 5.2 |
| **15** | **Tunisia** | 421.0 | 64.0 | 2.0 | 377.0 | 27.0 | 81.0 | 2.0 |
|  |  | 86.4 | 13.1 | 0.4 | 77.4 | 5.5 | 16.6 | 0.4 |
| **16** | **Iraq** | 324.0 | 176.0 | 19.0 | 453.0 | 64.0 | 1.0 | 1.0 |
|  |  | 62.4 | 33.9 | 3.7 | 87.3 | 12.3 | 0.2 | 0.2 |
| **17** | **Sudan** | 564.0 | 36.0 | 8.0 | 455.0 | 49.0 | 4.0 | 1.0 |
|  |  | 110.8 | 7.1 | 1.6 | 89.4 | 9.6 | 0.8 | 0.2 |

| **Table S3. Participants’ Occupation** | | | | | | | | |
| --- | --- | --- | --- | --- | --- | --- | --- | --- |
|  |  | **Regular employee** | **Shift employee** | **Freelancer** | **Do not work** | **Student** | **Left work because of COVID-19** | **Medical field** |
| **1** | **Egypt** | 42.0 | 16.0 | 11.0 | 28.0 | 334.0 | 8.0 | 30.0 |
|  |  | 9.0 | 3.4 | 2.3 | 6.0 | 71.2 | 1.7 | 6.4 |
| **2** | **Jordan** | 50.0 | 17.0 | 10.0 | 40.0 | 261.0 | 8.0 | 28.0 |
|  |  | 12.1 | 4.1 | 2.4 | 9.7 | 63.0 | 1.9 | 6.8 |
| **3** | **United Arab Emirates** | 179.0 | 22.0 | 19.0 | 70.0 | 95.0 | 16.0 | 6.0 |
|  |  | 44.0 | 5.4 | 4.7 | 17.2 | 23.3 | 3.9 | 1.5 |
| **4** | **Kuwait** | 140.0 | 18.0 | 16.0 | 60.0 | 36.0 | 18.0 | 142.0 |
|  |  | 32.6 | 4.2 | 3.7 | 14.0 | 8.4 | 4.2 | 33.0 |
| **5** | **Bahrain** | 101.0 | 15.0 | 15.0 | 80.0 | 193.0 | 7.0 | 27.0 |
|  |  | 23.1 | 3.4 | 3.4 | 18.3 | 44.1 | 1.6 | 6.2 |
| **6** | **Saudi Arabia** | 143.0 | 24.0 | 38.0 | 80.0 | 28.0 | 8.0 | 98.0 |
|  |  | 34.1 | 5.7 | 9.1 | 19.1 | 6.7 | 1.9 | 23.4 |
| **7** | **Oman** | 2.0 | 2.0 | 0.0 | 2.0 | 5.0 | 0.0 | 2.0 |
|  |  | 15.4 | 15.4 | 0.0 | 15.4 | 38.5 | 0.0 | 15.4 |
| **8** | **Qatar** | 5.0 | 4.0 | 0.0 | 1.0 | 9.0 | 2.0 | 1.0 |
|  |  | 22.7 | 18.2 | 0.0 | 4.5 | 40.9 | 9.1 | 4.5 |
| **9** | **Yemen** | 66.0 | 36.0 | 12.0 | 9.0 | 39.0 | 8.0 | 52.0 |
|  |  | 29.7 | 16.2 | 5.4 | 4.1 | 17.6 | 3.6 | 23.4 |
| **10** | **Syria** | 49.0 | 35.0 | 34.0 | 30.0 | 205.0 | 8.0 | 61.0 |
|  |  | 11.6 | 8.3 | 8.1 | 7.1 | 48.6 | 1.9 | 14.5 |
| **11** | **Palestine** | 50.0 | 20.0 | 3.0 | 19.0 | 274.0 | 8.0 | 25.0 |
|  |  | 12.5 | 5.0 | 0.8 | 4.8 | 68.7 | 2.0 | 6.3 |
| **12** | **Algeria** | 24.0 | 12.0 | 3.0 | 8.0 | 103.0 | 5.0 | 14.0 |
|  |  | 14.2 | 7.1 | 1.8 | 4.7 | 60.9 | 3.0 | 8.3 |
| **13** | **Morocco** | 38.0 | 16.0 | 5.0 | 6.0 | 75.0 | 3.0 | 3.0 |
|  |  | 26.0 | 11.0 | 3.4 | 4.1 | 51.4 | 2.1 | 2.1 |
| **14** | **Libya** | 243.0 | 25.0 | 12.0 | 51.0 | 101.0 | 6.0 | 82.0 |
|  |  | 46.7 | 4.8 | 2.3 | 9.8 | 19.4 | 1.2 | 15.8 |
| **15** | **Tunisia** | 82.0 | 24.0 | 8.0 | 15.0 | 319.0 | 8.0 | 31.0 |
|  |  | 16.8 | 4.9 | 1.6 | 3.1 | 65.5 | 1.6 | 6.4 |
| **16** | **Iraq** | 103.0 | 23.0 | 15.0 | 56.0 | 239.0 | 26.0 | 57.0 |
|  |  | 19.8 | 4.4 | 2.9 | 10.8 | 46.1 | 5.0 | 11.0 |
| **17** | **Sudan** | 36.0 | 15.0 | 10.0 | 16.0 | 400.0 | 7.0 | 25.0 |
|  |  | 7.1 | 2.9 | 2.0 | 3.1 | 78.6 | 1.4 | 4.9 |

| **Table S4. Participants’ education level and COVID-19 history.** | | | | | | | | |
| --- | --- | --- | --- | --- | --- | --- | --- | --- |
|  |  | **Education level** | | | | | **Did you or any related household have the corona virus infection** | |
|  |  | **Basic** | **Secondary** | **Higher education** | **Postgraduate studies** | **Other** | **yes** | **no** |
| **1** | **Egypt** | 9.0 | 31.0 | 391.0 | 37.0 | 1.0 | 85.0 | 384.0 |
|  |  | 1.9 | 6.6 | 83.4 | 7.9 | 0.2 | 18.1 | 81.9 |
| **2** | **Jordan** | 2.0 | 43.0 | 339.0 | 29.0 | 1.0 | 2.0 | 412.0 |
|  |  | 0.5 | 10.4 | 81.9 | 7.0 | 0.2 | 0.5 | 99.5 |
| **3** | **United Arab Emirates** | 1.0 | 75.0 | 233.0 | 93.0 | 5.0 | 23.0 | 384.0 |
|  |  | 0.2 | 18.4 | 57.2 | 22.9 | 1.2 | 5.7 | 94.3 |
| **4** | **Kuwait** | 2.0 | 37.0 | 236.0 | 149.0 | 6.0 | 53.0 | 377.0 |
|  |  | 0.5 | 8.6 | 54.9 | 34.7 | 1.4 | 12.3 | 87.7 |
| **5** | **Bahrain** | 4.0 | 109.0 | 305.0 | 16.0 | 4.0 | 72.0 | 366.0 |
|  |  | 0.9 | 24.9 | 69.6 | 3.7 | 0.9 | 16.4 | 83.6 |
| **6** | **Saudi Arabia** | 4.0 | 50.0 | 277.0 | 83.0 | 5.0 | 74.0 | 345.0 |
|  |  | 1.0 | 11.9 | 66.1 | 19.8 | 1.2 | 17.7 | 82.3 |
| **7** | **Oman** |  | 5.0 | 5.0 | 3.0 |  | 2.0 | 11.0 |
|  |  | 0.0 | 38.5 | 38.5 | 23.1 | 0.0 | 15.4 | 84.6 |
| **8** | **Qatar** | 1.0 | 9.0 | 9.0 | 3.0 | 0.0 | 1.0 | 21.0 |
|  |  | 4.5 | 40.9 | 40.9 | 13.6 | 0.0 | 4.5 | 95.5 |
| **9** | **Yemen** | 44.0 | 37.0 | 113.0 | 26.0 | 2.0 | 93.0 | 129.0 |
|  |  | 19.8 | 16.7 | 50.9 | 11.7 | 0.9 | 41.9 | 58.1 |
| **10** | **Syria** | 27.0 | 48.0 | 287.0 | 57.0 | 3.0 | 169.0 | 253.0 |
|  |  | 6.4 | 11.4 | 68.0 | 13.5 | 0.7 | 40.0 | 60.0 |
| **11** | **Palestine** | 1.0 | 6.0 | 29.0 | 349.0 | 14.0 | 21.0 | 378.0 |
|  |  | 0.3 | 1.5 | 7.3 | 87.5 | 3.5 | 5.3 | 94.7 |
| **12** | **Algeria** | 1.0 | 4.0 | 131.0 | 31.0 | 2.0 | 46.0 | 123.0 |
|  |  | 0.6 | 2.4 | 77.5 | 18.3 | 1.2 | 27.2 | 72.8 |
| **13** | **Morocco** | 4.0 | 13.0 | 77.0 | 51.0 | 1.0 | 7.0 | 139.0 |
|  |  | 2.7 | 8.9 | 52.7 | 34.9 | 0.7 | 4.8 | 95.2 |
| **14** | **Libya** | 47.0 | 113.0 | 346.0 | 13.0 | 1.0 | 23.0 | 497.0 |
|  |  | 9.0 | 21.7 | 66.5 | 2.5 | 0.2 | 4.4 | 95.6 |
| **15** | **Tunisia** | 3.0 | 68.0 | 343.0 | 71.0 | 2.0 | 7.0 | 480.0 |
|  |  | 0.6 | 14.0 | 70.4 | 14.6 | 0.4 | 1.4 | 98.6 |
| **16** | **Iraq** | 11.0 | 138.0 | 270.0 | 84.0 | 16.0 | 125.0 | 394.0 |
|  |  | 2.1 | 26.6 | 52.0 | 16.2 | 3.1 | 24.1 | 75.9 |
| **17** | **Sudan** | 2.0 | 75.0 | 387.0 | 41.0 | 4.0 | 48.0 | 461.0 |
|  |  | 0.4 | 14.7 | 76.0 | 8.1 | 0.8 | 9.4 | 90.6 |
