# Supplementary material for: Influence of COVID-19 on lifestyle behaviors in the Middle East and North Africa Region: a survey of 5896 individuals
Source: J Transl Med. 2021 Mar 30;19:129. doi: 10.1186/s12967-021-02767-9 (PMC8008335; doi:10.1186/s12967-021-02767-9)
Supplement: Supplementary file 3 — Additional file 3. Survey Development. [file 12967_2021_2767_MOESM3_ESM.docx]

**Questionnaire development**

Literature research was conducted in order to develop the survey questions. Some questions were adapted from similar published studies covering the same topics and others were developed based on literature review and recent studies (Table SI). An online Zoom meeting was conducted with expertise in which they provided their feedback to improve the survey. Based on the feedback several questions were modified (we deleted the psychological impact domain and health concerns domain). Finally, the pilot study was conducted (N=447). Cronbach's Alpha was calculated for each domain Tables SII, SIII, SIV and SV.

To calculate the sample size for the pilot study, the following equation was used.

$\boldsymbol{n}_{\boldsymbol{0}}\boldsymbol{=}\frac{\boldsymbol{z}^{\boldsymbol{2}}\boldsymbol{\times p(1-p)}}{\boldsymbol{e}^{\boldsymbol{2}}}$

|  | | |
| --- | --- | --- |
| $\boldsymbol{n}_{\boldsymbol{0}}$ | - | Sample size, which was estimated |
| $\boldsymbol{z}^{\boldsymbol{2}}$ | - | The selected critical value of the desired level of confidence or risk |
| $\boldsymbol{p}$ | - | The estimated proportion of an attribute that is present in  the population or maximum variability of the population |
| $\boldsymbol{e}$ | - | The desired level of precision or margin of error |

In our case, since the population number is unknown.

| $\boldsymbol{n}_{\boldsymbol{0}}$ | - | ? |
| --- | --- | --- |
| $\boldsymbol{z}^{\boldsymbol{2}}$ | - | 95% confidence level (The value of (1-α) in Standard Normal Distribution ***z***-table, which is 1.96 for 95%) |
| $\boldsymbol{p}$ | - | 50% variability of the population (which is maximum) |
| $\boldsymbol{e}$ | - | 5% margin of error |

Put the value in the given formula-

$$\boldsymbol{n}_{\boldsymbol{0}}\boldsymbol{=}\frac{\left( \boldsymbol{1.96} \right)^{\boldsymbol{2}}\boldsymbol{\times0.5}\left( \boldsymbol{1-0.5} \right)}{\left( \boldsymbol{0.05} \right)^{\boldsymbol{2}}}\boldsymbol{=}\boldsymbol{384}$$

**Table SI. Sources used to develop the survey questions available in (Supplementary data, questionnaire).**

| Question (s) | Source |
| --- | --- |
| **Daily eating habits** | |
| Q12^α^, Q18^α^, Q20^α^ | (1) |
| Q13^β^ | (1–3) |
| Q14^β^, Q15^β^, Q16^β^, Q17^β^, Q19^β^, Q24^β^ | (1) |
| Q21^€^ | (4) |
| Q22^β^ | (1,5) |
| Q23^β^ | (5,6) |
| Q25^£^ |  |
| Q26^€^ | (7) |
| **Lifestyle** | |
| Q27^β^ | (1,5) |
| Q28^β^ | (8) |
| Q29^β^, Q30^β^ | (1,6) |
| Q31^β^, Q32^β^ | (1) |
| Q33^β^ | (3) |
| Q34^€^ | (9) |
| Q35^β^ | (10) |
| Q36^€^ | (11) |
| β – implemented with modification from another survey  α – implemented from another survey  € – created based on information from published articles  £ – explanatory question | |

**Table SII. Cronbach's Alpha measures summary**

| items | Numbers of questions in each item | Cronbach's Alpha | Cronbach's Alpha Based on Standardized Items |
| --- | --- | --- | --- |
| daily eating habits | 28 | 0.729 | 0.807 |
| lifestyle | 18 | 0.697 | 0.658 |
| Total items | 46 | 0.786 | 0.822 |

**Table SIII: measuring *daily eating habits* as an induvial domain.**

| **Daily eating habits** | **Cronbach's Alpha** |
| --- | --- |
| Did you change your eating habits during confinement? | .728 |
| Did your weight change from before confinement? | .726 |
| How many times do you eat fruits and vegetables per week before | .719 |
| How many times do you eat fruits and vegetables per week after | .723 |
| How many times do you eat carbohydrates per day before | .715 |
| How many times do you eat carbohydrates per day after | .722 |
| How many times do you eat meats and poultry per week before | .708 |
| How many times do you eat meats and poultry per week after | .712 |
| How many times do you eat seafood per week before | .718 |
| How many times do you eat seafood per week before | .725 |
| How many litres (L) of water do you drink per day after | .724 |
| How many litres (L) of water do you drink per day before | .725 |
| How many dairy products do you consumer per day before | .716 |
| How many dairy products do you consumer per day after | .720 |
| How many eggs do you consume per week before? | .717 |
| How many eggs do you consume per week after? | .721 |
| How many teaspoons of sugar do you consume per day after | .718 |
| How many teaspoons of sugar do you consume per day before | .721 |
| How many snacks do you consume per week before? | .719 |
| How many snacks do you consume per week after? | .724 |
| How many times do you eat fast food per week before? | .716 |
| How many times do you eat fast food per week after? | .726 |
| What are drinks do you consume per day before? | .743 |
| What are drinks do you consume per day after | .745 |
| How many cups of drinks do you consume per day before? | .717 |
| How many cups of drinks do you consume per day after? | .720 |
| What are the dietary supplements do you consume per day before? | .718 |
| What are the dietary supplements do you consume per day after | .718 |

**Table SIV: measuring *lifestyle* as an induvial domain.**

| **lifestyle** | **Cronbach's Alpha** |
| --- | --- |
| How many times do you smoke per day before? | .692 |
| How many times do you smoke per day after | .705 |
| How many hours do you sleep per day before? | .695 |
| How many hours do you sleep per day after? | .708 |
| How many times do you practise physical activity per week Before? | .688 |
| How many times do you practise physical activity per week After? | .696 |
| How many minutes do you spend per each exercise before? | .696 |
| How many minutes do you spend per each exercise after | .714 |
| Before confinement, what were your physical activities | .694 |
| During confinement, what are your physical activities? | .694 |
| How many hours do you spend watching TV per day before? | .676 |
| How many hours do you spend watching TV per day after? | .687 |
| How many hours do you spend on social media per day before? | .651 |
| How many hours do you spend on social media per day after? | .657 |
| How many hours do you spend on the internet to (study/work) per day before? | .667 |
| How many hours do you spend on the internet to (study/work) per day after | .675 |
| How many hours do you spend with your family before? | .659 |

In general, Cronbach's alpha coefficients of at least 0.6 are thought to be indicative of good reliability (12). Also, according to Taber, Cronbach's alpha in the range of (0.67–0.87) is reasonable (13)

**Table SV: measuring the total survey items.**

| **Daily eating habits** | **Cronbach's Alpha** |
| --- | --- |
| Did you change your eating habits during confinement? | .785 |
| Did your weight change from before confinement? | .784 |
| How many times do you eat fruits and vegetables per week before | .780 |
| How many times do you eat fruits and vegetables per week after | .782 |
| How many times do you eat carbohydrates per day before | .777 |
| How many times do you eat carbohydrates per day after | .781 |
| How many times do you eat meats and poultry per week before | .773 |
| How many times do you eat meats and poultry per week after | .775 |
| How many times do you eat seafood per week before | .780 |
| How many times do you eat seafood per week before | .784 |
| How many litres (L) of water do you drink per day after | .781 |
| How many litres (L) of water do you drink per day before | .783 |
| How many dairy products do you consumer per day before | .778 |
| How many dairy products do you consumer per day after | .781 |
| How many eggs do you consume per week before? | .779 |
| How many eggs do you consume per week after? | .781 |
| How many teaspoons of sugar do you consume per day after | .778 |
| How many teaspoons of sugar do you consume per day before | .781 |
| How many snacks do you consume per week before? | .780 |
| How many snacks do you consume per week after? | .783 |
| How many times do you eat fast food per week before? | .779 |
| How many times do you eat fast food per week after? | .785 |
| What are drinks do you consume per day before? | .804 |
| What are drinks do you consume per day after | .804 |
| How many cups of drinks do you consume per day before? | .779 |
| How many cups of drinks do you consume per day after? | .781 |
| What are the dietary supplements do you consume per day before? | .776 |
| What are the dietary supplements do you consume per day after | .777 |
| **Lifestyle** |  |
| How many times do you smoke per day before? | .784 |
| How many times do you smoke per day after | .788 |
| How many hours do you sleep per day before? | .784 |
| How many hours do you sleep per day after? | .787 |
| How many times do you practise physical activity per week Before? | .783 |
| How many times do you practise physical activity per week After? | .786 |
| How many minutes do you spend per each exercise before? | .784 |
| How many minutes do you spend per each exercise after | .789 |
| Before confinement, what were your physical activities | .785 |
| During confinement, what are your physical activities? | .786 |
| How many hours do you spend watching TV per day before? | .782 |
| How many hours do you spend watching TV per day after? | .784 |
| How many hours do you spend on social media per day before? | .773 |
| How many hours do you spend on social media per day after? | .774 |
| How many hours do you spend on the internet to (study/work) per day before? | .778 |
| How many hours do you spend on the internet to (study/work) per day after | .780 |
| How many hours do you spend with your family before? | .777 |

References

1. Di Renzo L, Gualtieri P, Pivari F, Soldati L, Attinà A, Cinelli G, et al. Eating habits and lifestyle changes during COVID-19 lockdown: an Italian survey. J Transl Med. 2020 Jun 8;18(1):229.

2. Fernandez-Rio J, Cecchini JA, Mendez-Gimenez A, Carriedo A. Weight changes during the COVID-19 home confinement. Effects on psychosocial variables. Obes Res Clin Pract. 2020 Jul 1;14(4):383–5.

3. Matsungo TM, Chopera P. Effect of the COVID-19-induced lockdown on nutrition, health and lifestyle patterns among adults in Zimbabwe. BMJ Nutr Prev Health [Internet]. 2020 Dec 1 [cited 2021 Jan 26];3(2). Available from: https://nutrition.bmj.com/content/3/2/205

4. Butler MJ, Barrientos RM. The impact of nutrition on COVID-19 susceptibility and long-term consequences. Brain Behav Immun. 2020 Jul 1;87:53–4.

5. Constant A, Conserve DF, Gallopel-Morvan K, Raude J. Socio-Cognitive Factors Associated With Lifestyle Changes in Response to the COVID-19 Epidemic in the General Population: Results From a Cross-Sectional Study in France. Front Psychol [Internet]. 2020 [cited 2021 Jan 26];11. Available from: https://www.frontiersin.org/articles/10.3389/fpsyg.2020.579460/full

6. Ammar A, Brach M, Trabelsi K, Chtourou H, Boukhris O, Masmoudi L, et al. Effects of COVID-19 Home Confinement on Eating Behaviour and Physical Activity: Results of the ECLB-COVID19 International Online Survey. Nutrients. 2020 Jun;12(6):1583.

7. Adams KK, Baker WL, Sobieraj DM. Myth Busters: Dietary Supplements and COVID-19. Ann Pharmacother. 2020 Aug 1;54(8):820–6.

8. Gupta R, Grover S, Basu A, Krishnan V, Tripathi A, Subramanyam A, et al. Changes in sleep pattern and sleep quality during COVID-19 lockdown. Indian J Psychiatry. 2020;62(4):370–8.

9. Cinelli M, Quattrociocchi W, Galeazzi A, Valensise CM, Brugnoli E, Schmidt AL, et al. The COVID-19 social media infodemic. Sci Rep. 2020 Oct 6;10(1):16598.

10. Narayanan L, Pandit M, Basu S, Karmakar A, Bidhan V, Kumar H, et al. Impact of Lockdown due to COVID-19 Outbreak : Lifestyle Changes and Public Health Concerns in India. 2020 Jun 10 [cited 2021 Jan 26]; Available from: https://www.preprints.org/manuscript/202006.0129/v1

11. Lebow JL. Family in the Age of COVID-19. Fam Process. 2020 Jun;59(2):309–12.

12. Knapp TR. Focus on Psychometrics. Coefficient alpha: Conceptualizations and anomalies. Research in Nursing & Health. 1991;14(6):457-60.

13. Taber KS. The Use of Cronbach’s Alpha When Developing and Reporting Research Instruments in Science Education. Research in Science Education. 2017;48(6):1273-96.
